# Promoting evidence informed policy making in Nigeria: a review of the maternal, newborn and child health policy development process 

**DOI:** 10.15171/hpp.2017.33

**Published:** 2017-09-26

**Authors:** Chigozie Jesse Uneke, Issiaka Sombie, Namoudou Keita, Virgil Lokossou, Ermel Johnson, Pierre Ongolo-Zogo, Henry Chukwuemeka Uro-Chukwu

**Affiliations:** ^1^Knowledge Translation Platform, African Institute for Health Policy & Health Systems Studies, Ebonyi State University, PMB 053 Abakaliki, Nigeria; ^2^Organisation Ouest Africaine de la Santé, 175, avenue Ouezzin Coulibaly, 01 BP 153 Bobo-Dioulasso 01, Burkina Faso; ^3^Hopital Central Yaounde, CDBPH Lawrence VERGNE Building 2nd Floor, Avenue Henry Dunant Messa, Yaoundé, Cameroon

**Keywords:** Maternal, Newborn, Child, Evidence informed, Policymaking, Policy development

## Abstract

**Background:** There is increasing recognition worldwide that health policymaking process should be informed by best available evidence. The purpose of this study was to review the policy documents on maternal, newborn and child health (MNCH) in Nigeria to assess the extent evidence informed policymaking mechanism was employed in the policy formulation process.

** Methods:** A comprehensive literature search of websites of the Federal Ministry of Health(FMOH) Nigeria and other related ministries and agencies for relevant health policy documents related to MNCH from year 2000 to 2015 was undertaken. The following terms were used interchangeably for the literature search: maternal, child, newborn, health, policy, strategy,framework, guidelines, Nigeria.

**Results:** Of the 108 policy documents found, 19 (17.6%) of them fulfilled the study inclusion criteria. The policy documents focused on the major aspects of maternal health improvements in Nigeria such as reproductive health, anti-malaria treatment, development of adolescent and young people health, mid wives service scheme, prevention of mother to child transmission of HIV and family planning. All the policy documents indicated that a consultative process of collection of input involving multiple stakeholders was employed, but there was no rigorous scientific process of assessing, adapting, synthesizing and application of scientific evidence reported in the policy development process.

** Conclusion:** It is recommended that future health policy development process on MNCH should follow evidence informed policy making process and clearly document the process of incorporating evidence in the policy development.

## Introduction

Evidence-informed policy-making (EIP) is characterized by the systematic and transparent access to, and appraisal of, evidence as an input into policy-making.^1,2^ The National Institute for Health and Clinical Excellence (NICE)^3^ described evidence as either scientific or colloquial. The NICE report indicates that scientific evidence is the evidence arising from explicit, systemic, and replicable scientific and social scientific methods.^3^ Colloquial evidence complements scientific evidence and may come from expert testimony, evidence about values, practical considerations and the interests of specific groups.^3^

Evidence from scientific research has been consistently shown to be very reliable in the development and implementation of health policy.^4^ However, the lack of effective use of research evidence in policy-making continues to be a major challenge in most low- and middle-income countries (LMICs). In a previous report Oxman and colleagues,^5^ noted that a poorly-informed decision-making is one of the reasons why many LMICs failed to meet the health millennium development goals (MDGs).

With a population of over 160 million, Nigeria’s health system was ranked 187th out of 191 member states by the World Health Organization (WHO) in 2000.^6^ At an estimated 350 US dollars per capita annually, Nigeria ranks 152 out of 187 countries according to the United Nations Human Development Index (HDI) 2014 report in terms of per capita income.^7^ Due to the weak health systems, maternal and child health status in Nigeria is poor. With approximately 2.5% of the world’s population, Nigeria is reportedly having more than 10% of all under-5 and maternal deaths.^8,9^ More than 1 million newborn, infant, and child deaths and more than 50 000 maternal deaths are recorded every year in the country.^9,10^

The main causes of maternal mortality in Nigeria are: haemorrhage (23%), infection (17%), unsafe abortion (11%), obstructed labour (11%) and toxaemia/eclampsia/hypertension.^8-10^ Other factors underlying maternal mortality include lack of awareness about complications in pregnancy and on the need to seek medical intervention early.^8,10^ Recent reports have indicated that under 5 mortality ratio (U5MR) in Nigeria declined from 201 per 1000 live births in 2003^11^to 117 per 1000 live births in 2013.^12^Also the national maternal mortality ratio (MMR) declined from 800/100 000 in 2003^12^ to 576/100 000 in 2013.^13^ This reduction in MMR and U5MR could be attributed to the various maternal, newborn and child health (MNCH) intervention programmes and policies.

Studies appraising health policy documents regarding the use of research evidence in the policy development process are scarce in LMICs. The few available studies were conducted in high income countries with robust health systems and well-established evidence-informed policymaking process.^14,15^ In this article we report the outcome of a review of Nigerian MNCH policy documents, to assess the extent evidence informed policymaking process was employed in their formulation.

## Methods

A comprehensive literature search and identification of relevant Nigeria health policy documents on health policy related to MNCH from year 2000 to 2015 were undertaken. This was the main study inclusion criteria. We restricted the search to documents published from 2000-2015 because the initial search for health policy documents relevant to MNCH online which were published before year 2000 could not yield reasonable number of documents for 2 main reasons: (*i*) Most of the policy documents produced before the year 2000 have been updated and revised, and it is only these updated and revised versions that are made available electronically; (*ii*) Most of the health policy documents produced in the 1990s are available only in hard copies and so no electronic version exists. No software was used for the data extraction. The following terms were used interchangeably for the literature search: *maternal, child, newborn, health, policy, strategy, framework, guidelines, Nigeria.*

The websites of the Federal Ministry of Health (FMOH) Nigeria and other related ministries, agencies, departments, units, health service providers, non-governmental organizations (NGOs) relevant to MNCH were searched. These included National Planning Commission, National Primary Health Care Development Agency (NPHCDA), partnership for reviving routine immunization in northern Nigeria-maternal, newborn & child health (PRRINN-MNCH), World Bank and African Health Observatory. Additional searches were also conducted using the Google Scholar for health policy documents related to MNCH in Nigeria from 2000-2015. The Policy Information Platform Nigeria (http://www.pipnigeria.org), a repository for strengthening the access to and utilization of research evidence in health policymaking process was also searched.

The profiles of organizations that published the reviewed policy documents and the relationships between these organizations are shown in Table 1. The FMOH is the only organ of the Nigerian federal government that is mandated by law to produce national health policy documents. Other organizations can only produce guidelines, frameworks and protocols that are mainly derived from existing national health policies produced by the FMOH.

**Figure 1:**
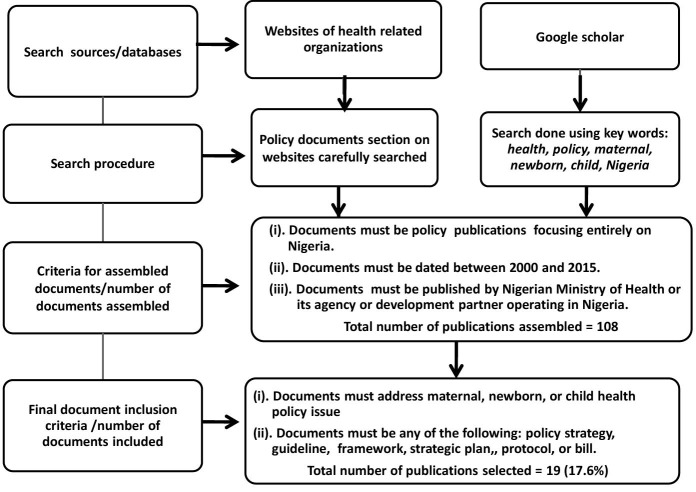
Flowchart of publication identification and selection process.

The publications identification and selection process is outlined in the flow chart shown in Figure 1. The main study inclusion criteria included:

(i) Documents must be policy publications focusing entirely on Nigeria.

(ii) Documents must be dated between 2000 and 2015.

(iii) Documents must be published by Nigerian Ministry of Health or its agency or development partner operating in Nigeria.

(iv) Documents must address maternal, newborn, or child health policy issue.

(vi) Documents must be any of the following: policy strategy, guideline, framework, strategic plan, protocol, or bill.

We reviewed the policy documents that fulfilled the study inclusion criteria for the following information:

(i) Reference to research evidence used if any, and indicated by citation of the research publication in the document;

(ii) the process of the production of research evidence used in the development of the policy documents;

(iii) the mechanisms and systems in place for knowledge transfer and use of health research findings in MNCH;

(vi) the barriers and facilitators with respect to researcher - policy makers’ interface, existing systems of monitoring and evaluation of knowledge transfer and use of research findings in the policy development process;

(v) funding for health research in MNCH related to the policy development.

Non-policy documents, and policy documents that were not based on MNCH were excluded. Document validity was assessed based on the study inclusion and exclusion criteria.

## Results

A total of 108 policy related documents were found. Of these 108 documents, 19 (17.6%) of them were identified as health policy documents that specifically focused on MNCH. These 19 health policy documents were reviewed according to the year of publication; title of policy document; document source/publisher; main focus of policy; main research evidence/ publication informing the policy cited in document; and process employed in the policy development. The policy documents were then classified into maternal health^16-23^ (Table 2), newborn health^10,12,24-26^ (Table 3), and child health^27-32^ (Table 4).

The policy documents reviewed neither reported the process of production of evidence used in the development nor indicated the mechanisms in place for knowledge transfer and use of health research findings. The policies did not also provide any information on the barriers and facilitators of researcher - policy makers’ interface. No description of monitoring and evaluation of knowledge transfer and use of research findings in the policy development was provided. There was also no information provided regarding funding for health research in MNCH.

The profile and characteristics of policy documents are presented in Table 2. Eight key policy documents were identified.^16-23^ These policy documents focused on the major aspects of maternal health improvements in Nigeria such as reproductive health, prevention of mother to child transmission of HIV and family planning. Of these eight policy documents, six^18-23^ had no single publication cited in the documents. The remaining two^16,17^ cited non-research-based publication, but made reference to other existing policy documents (Table 2). All the policy documents noted that a consultative process involving various groups of stakeholders at various levels was employed. There was no rigorous scientific process of assessing, adapting, synthesizing and application of high quality scientific evidence reported in the policy development process.

The five-identified newborn health policy documents^10,12,24-26^ focused on key health issues related to improvement of newborn health (Table 3). These included infant and child feeding, integrated MNCH, kangaroo training guidelines for low birth weight babies, and saving one million lives initiative (Table 3). No publications were cited in two of the policy documents.^10,24^ The remaining three policy documents^12,25,26^ cited some scientific publications from which the policy document contents were derived. The policy documents with scientific citations were produced by the Ministry of Health in conjunction with development partners such as World Bank and PRRINN-MNCH initiative.^25^ Furthermore the other documents with scientific citation were the more recent policy documents of 2015^12,26^ (Table 3).

Of the 6 policy documents identified associated with child health in Nigeria,^27-32^ only that produced by NPHCDA^29^ which is the Minimum Standards for Primary Health Care in Nigeria contained scientific citations (Table 4). The National Immunization Strategic Plan^30^ produced by NPHCDA made reference to other policy documents but cited no research-based publication. Both NPHCDA and FMOH developed the documents in conjunction with development partners and various professional health associations and stakeholders (Table 4).

It was noted in all the policy documents that a highly consultative process was employed involving various groups of stakeholders at various levels. The stakeholders engaged in the process included researchers, practitioners, development partners, professional health associations, civil society organizations, non-governmental organizations etc.

## Discussion

Findings from this study suggest that the FMOH has made remarkable efforts in the last 15 years to produce and implement health policies that have contributed to the improvement of MNCH outcomes in Nigeria.^13^ No description of process of evidence acquisition was provided in most of the policy documents reviewed and scientific references were not cited in the documents. However, because of the involvement of multiple stakeholders, the use of some sort of evidence may not be ruled out of the policy development process. Among the key stakeholders involved in the policy development were members of the Nigeria Medical Association (NMA) and Pharmaceutical Society of Nigeria (PSN). Most of the members of NMA (http://nationalnma.org/) and PSN (http://psnnational.org/) are both health practitioners and committed researchers. A previous report has shown that when researchers are involved in policy development process they are more likely to make contributions from scientific evidence.^33^

The recommendations and focus of the policy documents clearly showed that the evidence used in their development has roots from scientific discoveries. For instance, the National Reproductive Health Policy and Strategy published by FMOH^16^ has guidelines designed to achieve increase access to qualitative and affordable MNCH services. Some earlier scientific research from Nigeria on maternal health from 1996-1999 provided evidence supporting the guidelines outlined in the policy document.^34,35^ Similarly, the FMOH National Guidelines for the Prevention of Mother to Child Transmission of HIV (PMTCT),^22^ cited no scientific publications but its guidelines are consistent with recommendations from scientific research on PMTCT undertaken earlier in Nigeria.^36^ This is also applicable to an earlier study^37^ that informed the National family Planning/Reproductive Health Service Protocols and Policy Guidelines and Standard Practice.

Unlike the more recently published Nigeria’s Call to Action to Save Newborn Lives,^26^ by FMOH which cited some scientific publications, the 2 earlier policy documents^10,24^ associated with newborn health cited no research-based publications. It is note-worthy that most of the recommendations in these policies which are designed to improve newborn health are supported by findings from some research publications undertaken in Nigeria before year 2007.^34,35,37^

The more recent World Bank policy document^12^ on Saving One Million Lives Initiative in Nigeria, developed through a highly consultative process cited some key references on which the policy was derived. It therefore appeared that until recently, scientific publications are not usually cited in FMOH policy documents. Of the four key policy documents on child health (Table 4), only the Minimum Standards for Primary Health Care in Nigeria published by NPHCDA,^29^ cited research publications. This confirmed that earlier policy documents published by the FMOH rarely contained scientific publications citation. The contents of the policy documents however, indicated the use of research evidence in the development of the policies derived from previous studies.^36,37^

### Study limitation

This review has 2 main limitations. First, we acknowledge that the number of policy documents included in this review may not have been sufficient. We were handicapped by the fact that many of the Nigerian health policy documents as in other LMICs are not available online. The second limitation was our inability to assess the extent and the quality of scientific evidence used in the policy formulation. We recommend that future studies use the model introduced by Cheung and colleagues^14^ and Strehlenert and colleagues,^15^ who used a modified set of existing criteria derived from the logic of events theoretical framework, which conceptualizes the connection between policy determinants and outcomes.

## Conclusion

With several evidence-informed maternal and child health intervention programme and policies implemented in Nigeria, better MNCH outcomes are becoming evident. Incorporating evidence into policy making is neither a simple nor straightforward venture. According to Jones and Walsh,^38^ the integration of evidence into policy decision making is a complex process of multiple, frequently competing and/or intertwined sets of influences in which evidence plays just one of many roles. The implication of this is the need to promote evidence-to-policy link among policymakers and the establishment of mechanisms that will facilitate bridging of the gap between them and researchers. The CHSRF^39^ noted that decision makers need to demonstrate their readiness to work with researchers by committing time and effort to prepare for and receive research for decision making. It is also very important to improve the research capacity of policymakers to increase their likelihood of use of research evidence in policymaking.^40^ It is recommended that future policy development process should not only follow evidence informed policy making but also clearly document the process of incorporating the evidence into the policy development.

**Table 1:** Organizational profiles of organizations that published the policy documents included in the review

**Organization**	**Description of organization**	**Nature of relationship with other included organizations**	**Organization’s website**
FMOH	FMOH is the main national health policy making organ of Nigeria federal government.	FMOH has departments, agencies, parastatals and other arms of government. It collaborates with other organizations in policy process.	http://www.health.gov.ng/
NPHCDA	NPHCDA, is a parastatal of Nigeria’s Federal Ministry of Health with the mandate to: providing support to the PHC.	Collaborates with FMOH to control preventable diseases: improve access to basic health services: improve quality of care:	http://www.nphcda.gov.ng/
Partnership for reviving routine immunization in northern Nigeria-maternal, newborn & child health (PRRINN-MNCH)	The programme is managed by a consortium led by Health Partners International. Established to address these health issues in northern Nigeria.	Works with the government, to improve the quality and availability of maternal/child health services.	http://www.prrinn-mnch.org/
NPC	The NPC known as Ministry of Budget and National Planning. Advise on matters relating to national development and the economy.	In partnership with other national organizations to formulate and prepare national development plans.	http://www.nationalplanning.gov.ng/
WB	The WB is a vital source of financial and technical assistance to developing countries	Partners with FMOH and other arms of government to improve MNCH services	http://www.worldbank.org/en/country/nigeria

Abbreviations: NPHCDA, National Primary Health Care Development Agency; PHC, primary health care; FMOH, Federal Ministry of Health; PMTCT, Prevention of Mother to Child Transmission of HIV; PRRINN-MNCH, Partnership for reviving routine immunization in northern Nigeria-maternal, newborn & child health; NPC, National Planning Commission; WB, World Bank.

**Table 2:** Profile and characteristics of policy documents associated with maternal health in Nigeria

**No.**	**Document source/ year of publication/reference**	**Title of policy document**	**Main focus of policy**	**Citation of research evidence/ publication**	**Process employed in the policy development**
1.	FMOH, Abuja, 2001^16^	National reproductive health policy and strategy	Increase in access to qualitative and affordable maternal and child health services.	No research-based publication cited, other policy documents cited.	Through a highly consultative process involving stakeholders.
2.	FMOH, Abuja, 2002^17^	National reproductive health strategic framework and plan 2002–2006	Safe motherhood; family planning; sexually transmitted infections/HIV/AIDS; adolescent reproductive health/reproductive rights.	No research-based publication cited, other policy documents cited.	Developed by the Reproductive Health Division of FMOH.
3.	FMOH & NMVCD Abuja, 2005^18^	National Anti-malaria Treatment Policy	Essential antimalarial drugs, rational use of antimalarial drugs, management of antimalarial drug supply.	No publication was cited in the document.	Collection of input from stakeholders to develop policy.
4.	FMOH, Abuja, 2006^19^	The Nigeria National Blood Policy	Blood Transfusion Service in Nigeria; efficient National Blood Service and quality standards.	No publication was cited in the document.	Collection of input from stakeholders to develop policy.
5.	FMOH, Abuja, 2007^20^	National Policy on Health and Development of Adolescent and Young People in Nigeria	Sexual behaviour; reproductive health; drug abuse; career and employment.	No publication was cited in the document.	Through a highly consultative process involving stakeholders.
6.	PRRINN-MNCH, 2009^21^	Mid wives Service Scheme	Helps to address the critical shortage of skilled birth attendants in Northern Nigeria.	No publication was cited in the document.	Collection of input from stakeholders to develop scheme.
7.	FMOH, Abuja, 2010^22^	National Guidelines for the PMTCT	Prevention of HIV infection in women of reproductive age group and HIV transmission from HIV infected mothers to their infants.	No publication was cited in the document.	Collection of input from stakeholders to develop scheme.
8.	FMOH, Abuja, 2010^23^	National family Planning/Reproductive Health Service Protocols and Policy Guidelines and Standard Practice	Behaviour change communication; fertility awareness-based methods, contraception.	No publication was cited in the document.	Assembling of a task force to review a previously existing the policy.

Abbreviations: FMOH, Federal Ministry of Health; MNCH, maternal, newborn and child health; PMTCT, Prevention of Mother to Child Transmission of HIV; PRRINN-MNCH, partnership for reviving routine immunization in northern Nigeria-maternal, newborn & child health.

**Table 3:** Profile and characteristics of policy documents associated with newborn health in Nigeria

**No.**	**Document source/ year of publication/ Reference**	**Title of policy document**	**Main focus of policy**	**Citation of research evidence/ publication**	**Process employed in the policy development**
1.	FMOH Abuja, 2005^24^	National policy on infant and child feeding in Nigeria	Promote exclusive; promote timely introduction of complementary foods while breastfeeding.	No research-based publication cited, other policy documents cited.	Through a highly consultative process involving stakeholders.
2.	FMOH Abuja, 2007^10^	The integrated MNCH strategy	Provide guidance to develop operational/ implementation plans for MNCH.	No research-based publication cited, other policy documents cited.	Through a highly consultative process involving stakeholders.
3.	PRRINN-MNCH, 2011^25^	Saving Low Birth Weight Newborn Lives through KMC	Feasible and low-cost approach for managing LBW babies, and to reduce mortality.	Research-based publications cited	PRRINN-MNCH initiative worked with partners to develop training manual
4.	FMOH Abuja, 2015^26^	Nigeria’s call to action to save newborn lives	To end preventable newborn deaths, making life-saving interventions available.	Research-based publications cited	Through a highly consultative process involving stakeholders.
5.	World Bank, 2015^12^	Saving One Million Lives Initiative Nigeria	Priority to high impact, interventions eg., improving nutrition & immunization	Research-based publications cited	Through a highly consultative process involving stakeholders.

Abbreviations: FMOH, Federal Ministry of Health; MNCH, maternal, newborn and child health; KMC, Kangaroo Mother Care.

**Table 4:** Profile and characteristics of policy documents associated with child health in Nigeria

**No.**	**Document source/ year of publication/ Reference**	**Title of policy document**	**Main focus of policy**	**Citation of research evidence/ publication**	**Process employed in the policy development**
1.	National Planning Commission Abuja, 2005^22^	National Plan of Action on Food and Nutrition in Nigeria	To reduce the prevalence of malnutrition by alleviating poverty.	No publication was cited in the document.	Committee on Food and Nutrition worked with other stakeholders.
2.	FMOH Abuja, 2006^28^	National Child Health Policy	To implement the available evidence-based, cost-effective interventions in infants and children.	No publication was cited in the document.	FMOH worked with other stakeholders.
3.	NPHCDA, 2007^29^	Minimum Standards for Primary Health Care in Nigeria	To deliver PHC services, and consists of a set of health interventions and services that address health and health related problems.	Research-based publications cited	NPHCDA worked with other stakeholders.
4.	NPHCDA, 2013^30^	National Routine Immunization Strategic Plan	Guidelines on immunization safety, standards and specifications; and of surveillance systems.	No research-based publication cited, other policy documents cited.	NPHCDA worked with other stakeholders.
5.	FMOH, Abuja, 2010^31^	National Guidelines for paediatric HIV treatment and care	Provide evidence-based information on paediatric HIV and AIDS management and prevention.	No publication was cited in the document.	FMOH worked with other stakeholders.
6.	FMOH, Abuja, 2014^32^	The National Health Bill, 2014	Provide a framework for management of a national health system.	No publication was cited in the document.	FMOH worked with other stakeholders.

Abbreviations: NPHCDA, National Primary Health Care Development Agency; PHC, primary health care; FMOH, Federal Ministry of Health; MNCH, maternal, newborn and child health.

## Ethical approval

This review was a key component of the “*Moving Maternal, Neonatal and Child Health Evidence into Policy in West Africa”* (MEP) project undertaken by West African Health Organization (WAHO). Ethical clearance was obtained from the University Research Ethics Committee of Ebonyi State University Nigeria (the institution of the principal author).

## Competing interests

The authors declare no competing interests.

## Authors’ contributions

All authors participated in the design and development of the study. CJU, IS and PO reviewed the studies selected and agreed on the studies included. CJU drafted the manuscript, with contributions from IS, PO, NK and HCU. All authors made inputs to the final manuscript.

## Acknowledgements

This study was one of the outcomes of the “*Moving Maternal, Neonatal and Child Health Evidence into Policy in West Africa”* ​​(MEP) project undertaken by West African Health organization (WAHO) funded by International Development Research Centre (IDRC) Canada (Reference: IDRC 107892_001). Authors are grateful to all the policymakers, researchers and other stakeholders in MNCH in Nigeria who participated in this study.
